# Extremely Extensive Vascular Malformation Requires Special Preparation for Simple Dental Surgical Procedures—Case Report

**DOI:** 10.3390/dj13050217

**Published:** 2025-05-19

**Authors:** Natalia Muczkowska, Klaudia Masłowska, Agnieszka Adamska

**Affiliations:** Department of Oral Surgery, Medical University of Warsaw, 02-097 Warsaw, Poland; natalia.muczkowska@wum.edu.pl (N.M.); klaudia.maslowska@wum.edu.pl (K.M.)

**Keywords:** vascular malformations, cavernous haemangioma, oral surgery, tooth extraction

## Abstract

**Background/Objectives:** Vascular anomalies represent a complex group of conditions including vascular malformations and haemangiomas. Haemangiomas are benign tumours that have an endothelial origin. In contrast, vascular malformations are characterized by the abnormal dilation of vessels without proliferation. Depending on the extension of the disease, there is a higher risk of life-threatening haemorrhages that may occur during simple dental procedures. The aim of this case report is to present the interdisciplinary treatment for patients with venous malformation and to discuss the possible dental management of these patients. **Methods:** A 66-year-old male patient with an extensive venous malformation of the head and neck was referred for a tooth extraction. The venous malformation involved lips, buccal mucosa, tongue, and floor of the oral cavity. Its proximity to the tooth requiring extraction was associated with a high risk of severe bleeding. **Results:** Prior to the treatment, CBCT and CT scans were performed to confirm the extensions of the lesion and visualise its margins. Considering the possible risks related with venous malformation, the procedure consisted of tooth removal in a hospital setting with control over severe bleeding complications. **Conclusions:** The presence of an extensive vascular malformation in the head and neck region is burdened with a higher risk of haemorrhages during simple dental procedures. The radiological and clinical planning enables the choice of an accurate treatment strategy to avoid possible difficulties. In cases where such complications cannot be avoided, it is important to perform the treatment in a hospital setting with the cooperation of maxillofacial surgeons.

## 1. Introduction

Vascular malformations (VMs—vascular malformations) are a broad group of lesions that affect the vascular or lymphatic systems, featuring significant heterogeneity in the head and neck area. It has been established that pathogenesis is associated with the disrupted morphogenesis of the endothelium cells (ECs—endothelial cells) resulting in structural disorders defined by the abnormal dilation of vessels without cell proliferation. Most recently, ISSVA (ISSVA—International Society for the Study of Vascular Anomalies) classified vascular anomalies as “vascular tumours” (HM—haemangiomas) and “vascular malformations” according to the pathology’s biological characteristics and potentially unique vascular anomaly (PUVA) [1,2,3].

Haemangiomas are caused by proliferative changes in vascular ECs and represent the most common tumours in infancy, with an incidence ranging from 5 to 10% among one-year-old children. The prevalence of haemangiomas is significantly higher in females than in males (ratio: 3:1 to 5:1). The head and neck region accounts for 60% of all cases [4]. The aetiology of these lesions is not fully known. The suspected causes of HM development include angiogenic factors, tissue hypoxia, and hormonal disturbances. The main immunochemical marker that confirms the diagnosis of infantile haemangioma is GLUT-1 (GLUT-1—Glucose transporter-1) [3,4,5]. These mesenchymal neoplasms are classified as benign congenital hamartomas and occur in the first eight weeks of life with a characteristic rapid growth stage, which is followed by a gradual regression. A burn-like scar is left after the involution of an HM, which corresponds to fatty fibrous residues. Due to spontaneous involution, most haemangiomas require no treatment although a surgical correction of scars might be performed for aesthetics, especially those located in facial area [4,5,6].

Unlike haemangiomas, vascular malformations are present at birth and persist throughout life. Their classification is based on the type of vessel involved and hemodynamic features, including low-flow lesions (capillary, venous, and lymphatic) and high-flow lesions (AVMs—arterio-venous malformations). The combined term vascular malformations describes the lesions that share characteristics of multiple VMs. “Port wine stain” refers to capillary malformation, which occurs often in the head and neck area, especially along trigeminal nerve dermatomes. These lesions may involve the underlying lips, cheeks, and gingiva, causing gingival hypertrophy and chronic bleeding. Venous malformations, predominantly termed as “cavernous haemangiomas”, in contrast to haemangiomas, do not regress spontaneously and may include bone lesions. Usually, they manifest as soft subcutaneous masses in a blue-purple colour, which may infiltrate deeply along the fascial planes of the head and neck region [1,2,3,6].

Treatment of the venous malformation is determined by its location, organ dysfunctions, and functional impairment. The lesions located in the head and neck area are associated with a higher risk of a haemorrhage or ulceration, particularly during dental procedures. Treatment methods that might be implemented for low-flow VMs include sclerotherapy, embolization, steroid administration, laser photocoagulation therapy, and surgical resectioning. However, there are few papers concerning the dental approach in patients diagnosed with VMs that could simplify the treatment algorithms and avoid life-threatening consequences [6,7,8,9].

The aim of this paper is to present the case report of the dental management of a male patient diagnosed with venous malformation in the head and neck area and to discuss possible treatment methods from a dental perspective.

## 2. Case Report

In May 2024, a 66-year-old man was referred by a general dentist to the Department of Oral Surgery, Medical University in Warsaw for a surgical sanitation of an oral cavity.

According to the medical record, the patient was born with an extensive vascular malformation involving the body of the tongue, the floor of the mouth, and the subcutaneous tissue of the cheeks and neck. The main diagnosis consisted of venous malformation (“cavernous haemangioma”) of the neck involving adipose tissue and a lymphangioma, infiltrative type, located in the tongue. During the patient’s childhood, the previous treatment methods of vascular lesion included X-ray diathermy, implantation of radioactive gold grains, short-term alcohol injections into the tongue, and attempts of isolated coagulation. However, regression of the vascular lesions was not observed after any of the used therapies. Due to a steady growth of the tongue, a surgery was performed to wedge out part of the tongue shaft, using surgical pins to hold the tongue in the correct position. A biopsy of the vascular lesion in the neck area resulted in a venous blood sample, confirming the diagnosis of venous malformation.

**Figure 1:**
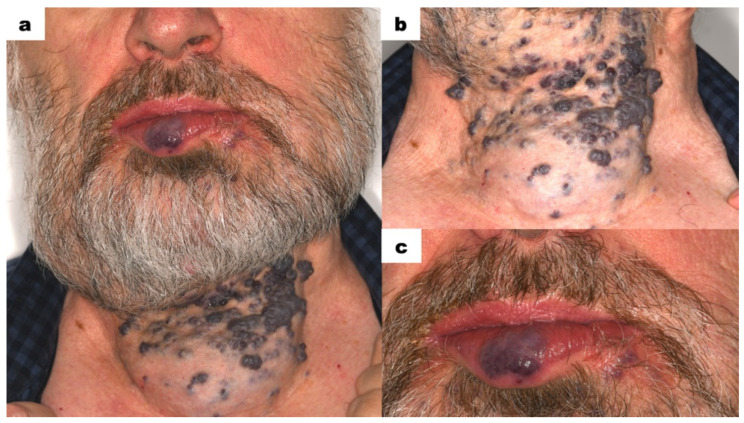
Extraoral photographs; (**a**) asymmetry in the submandibular region and a widespread neck malformation, with visible purple and navy-blue nodules along the midline of the neck; (**b**) malformation in the subcutaneous tissue of the neck; (**c**) purple elevation at the central point of the vermilion border—vascular malformation of the lower lip.

The patient’s face was markedly asymmetrical, particularly in the lower facial region. Due to the presence of VMs, the right side of the mandible was enlarged compared to the left side (Figure 1a). The neck along the midline was significantly enlarged. On the neck, scattered, hard nodules of a dark purple colour were present (Figure 1b). These nodules did not blanch on pressure. Lymph nodes were non-painful and not palpable. A bluish-red nodule was visible in the centre of the lower lip (Figure 1c). The lesion was not movable in relation to the underlying tissues and did not disappear on pressure. The lower lip was slightly everted. Small pinpoint bluish-red spots were visible on the skin below the left corner of the mouth. Apart from the aforementioned changes, no oral dysfunctions or parafunctions were noted in the extraoral examination.

**Figure 2:**
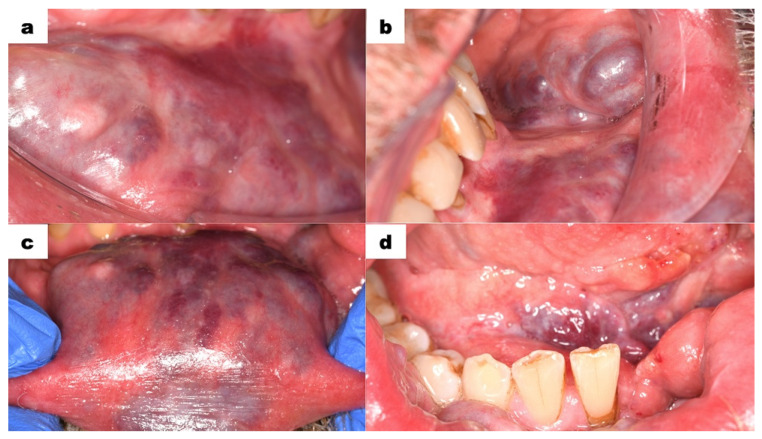
Intraoral photographs; (**a**) the flattening of the oral vestibule, the mucous membrane of the lower lip covered by purple and red elevations; (**b**) the left buccal mucosa shows visible purple nodules covering a wide area of the mucous membrane; (**c**) the lower lip, viewed from above, shows that the vascular malformation affects the entire thickness of the lower lip; (**d**) vascular malformation of the floor of the mouth, with a shortened tongue body, the patient is unable to elevate the tongue to the palate.

The intraoral examination was performed with extreme delicacy and caution, considering the presence of venous malformations in the oral mucosa. The use of retractors was minimized due to the high risk of tissue injury and bleeding. The vestibule at the level of the lower incisors was shallow. Elevated areas and thickening in shades of purple and red were observed (Figure 2a). The mucous membrane on the inner side of the lower lip and left cheek exhibited visible nodules in red, purple, and bluish colours (Figure 2b,c). On the right side, the lesions were less pronounced. The floor of the mouth was shallow, covered by mucous tissue in a dark purple colour. Hypertrophy of the sublingual folds was visible. The tongue showed significantly limited mobility. The patient’s speech was partially unclear. The intraoral examination showed poor oral hygiene, tooth 31 (FDI classification) was significantly mobile but non-painful on palpation. No discomfort was noted during vertical and horizontal percussion. Numerous teeth had composite restorations, some of which required replacement due to the inadequate sealing. Teeth 31 and 41 were markedly spaced apart and deviated from the midline. Teeth from 33 to 43 were in traumatic occlusion. Signs of excessive wear were observed in tooth 43. Tooth 47 had a primary carious lesion. Tooth 38 was not visible in the oral cavity.

**Figure 3:**
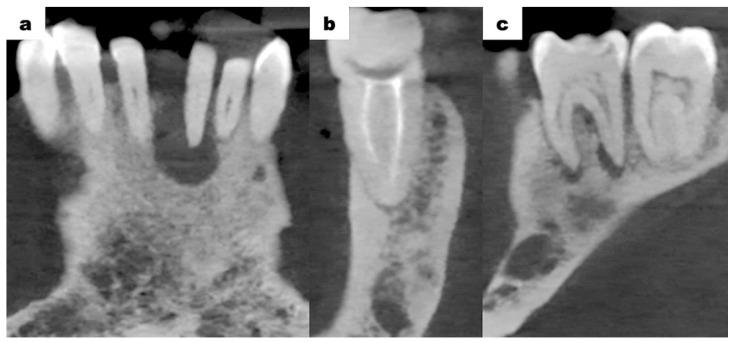
CBCT examination scans before initiation of treatment; (**a**) tooth 31—inflammatory change involving the entire root; (**b**) tooth 46—no trace of filling material in the root canals in the apical region, a suspected periapical lesion; (**c**) tooth 36—periapical inflammatory changes visible around both roots.

**Figure 4:**
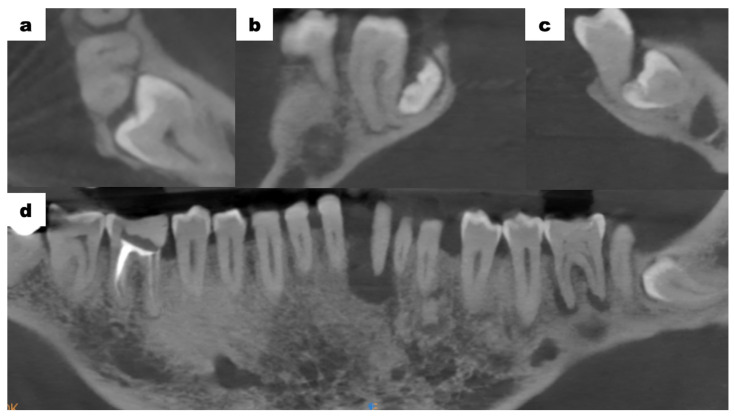
Mandibular CBCT examination scans before initiation of treatment; (**a**) an axial plane scan—tooth 38 retained in the horizontal, mesial-angular position; (**b**) a sagittal plane scan—tooth 37 distally exposed due to the adjacent position of tooth 38, no signs of root resorption; (**c**) a frontal plane scan—between tooth 37 and 38, visible potential plaque retention; (**d**) anteroposterior projection—bone thinning in the chin region.

Cone-beam computed tomography (CBCT—cone-beam computed tomography) scans of the mandible, which were provided by the patient, revealed bone resorption around tooth 31. The lateral surfaces of the apices of teeth 41 and 32 were adjacent to the periapical lesion of tooth 31 (Figure 3a). Tooth 46 was treated endodontically for a deficiency of filling material in the apical region of the roots (Figure 3b). Tooth 47 showed a carious lesion. Tooth 36 exhibited extensive periapical changes (Figure 3c). Tooth 38 was partially retained in the horizontal, mesial-angular position with no eruption potential. No inflammatory cysts were found around the crown of the retained tooth. There were no signs of resorption of tooth 37 (Figure 4a). However, the distal root surface of tooth 37 was exposed due to the adjacent position of tooth 38, enabling plaque retention. This might be a potential source of inflammation (Figure 4b). In the event of peri-coronal inflammation, tooth 38 will be eligible for an extraction in the hospital setting. CBCT scans revealed areas of bone thinning in the chin region, potentially due to irradiation treatment when the patient was at the developmental age (Figure 4c).

**Figure 5:**
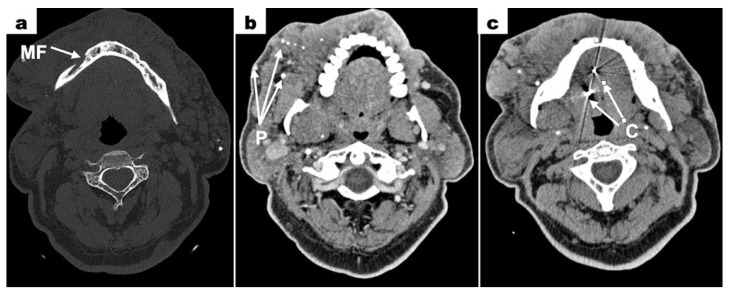
Contrast-enhanced CT scans; (**a**) lack of a defined boundary between the mental foramen and the vascular malformation mass (MF = mental foramen); (**b**) extensive areas of densification with calcifications in the blood vessels of the vascular malformation mass (P = phleboliths); (**c**) metallic foreign bodies in the projection of the tongue—clips used in a treatment to tether the tongue in childhood (C = clips).

Computed tomography (CT—computed tomography) scans revealed a bone defect measuring 7 × 4.5 mm surrounding the root of tooth 31. Within this area, a tissue mass measuring 12 × 9 mm was observed, partially enhanced after contrast administration. The left mandibular canal was enlarged. Contrast enhancement was noted around the left mental foramen (Figure 5a). The image confirmed the lack of a clear boundary between the mental foramen and the vascular malformation mass. This suggested the infiltration of the vascular malformation into the mental foramen only on the left side. Extensive areas of densification with calcifications were visible in the skin and subcutaneous tissue of the neck, chin, and cheeks (primarily the left cheek) (Figure 5b). Among these, dilated vessels were present, suggesting a large vascular malformation. Metallic foreign bodies were visible in the projection of the tongue, which showed heterogeneous enhancement after contrast administration. Two radiopaque spots were visible in the image—clips used in a treatment to tether the tongue in childhood (Figure 5c). The left submandibular gland was not visualized. A densification in the subcutaneous tissue of the right side of the neck, measuring 9 × 3 mm, was noted. No enlarged lymph nodes in the cervical region were seen.

**Figure 6:**
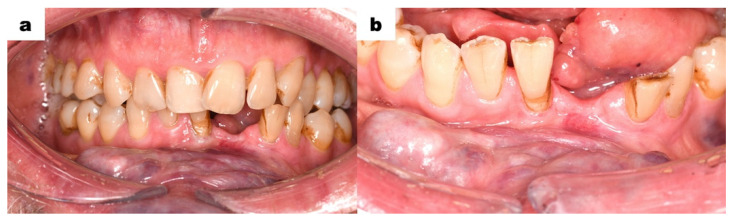
(**a**,**b**): Intraoral photographs 7 months after the extraction of tooth 31.

The treatment plan included the extraction of tooth 31, conservative removal of carious lesions, endodontic treatment, and oral hygienisation (oral hygiene management)**.** The surgery was performed in the antibiotic shield under local anaesthesia using lignocaine 2% with noradrenaline in a hospital setting. Tooth 31 was removed entirely. There was no increased bleeding from the tooth extraction socket. The lesion was curetted and submitted for histopathological examination. The cavity was rinsed with a Metronidazole solution and sutures were placed. Postoperatively, the patient was given an antibiotic (amoxicillin 875 mg/clavulanic acid 125 mg) every 12 h for 7 days. A follow-up visit was scheduled after 7 days of uneventful healing. No postoperative complications were noted. The follow-up 7 months after the procedure showed a properly healed alveolus after extraction (Figure 6a,b).

The histopathological examination result of the periapical lesion was the purulent granulomatous tissue. Regarding the clinical and radiological examinations, which revealed extensive vascular changes, it was concluded that a simple procedure such as a tooth extraction could result in an extensive haemorrhage. No postoperative complications after extraction of tooth 31 were observed. The sutures were removed after 7 days of uneventful healing. The patient did not report any complications such as bleeding or inflammation.

The patient was referred to hospital for further diagnostics and potential extraction of tooth 38. The patient will undergo conservative and endodontic treatment at a highly specialized referral centre. Due to the severity of the vascular lesion on the left side and the high risk of bleeding, the procedure will take place under general anaesthesia with at least two units of blood secured. Because the vessel supplying the vascular malformation could not be determined, bleeding from intraosseous vessels should be considered. Hospital conditions allow a response to the situation of a haemorrhage by transfusing blood, using coagulation forceps, and, in extreme cases, immediate segmental resectioning of the mandible.

The final diagnosis is difficult to establish when it comes to vascular changes. Based on consultations with maxillofacial surgeons, changing the diagnosis from a haemangioma to a vascular malformation was suggested. The fact that the patient has had the condition since birth supports the diagnosis of a vascular malformation. Additionally, it has grown rather than regressed with age. The vascular change is significant enough that none of the surgeons who have previously consulted the patient have undertaken treatment. There is suspicion that the vessel supplying such an extensive vascular lesion is very large, and its closure could lead to serious complications. The patient does not agree to invasive diagnostic procedures just to change the diagnosis, and an MRI cannot be performed due to the metal clips previously used to elevate the tongue.

## 3. Discussion

Vascular anomalies are heterogenous and involve a broad range of lesions associated with soft tissue and organ disorders. The ISSVA classification is widely accepted by both the clinical and scientific community and considered the gold standard for diagnosing and treating patients according to clinical findings and associated syndromes. This evolving field of study continues to expand as our understanding of the biology and genetics of vascular malformations and tumours advances [10,11]. The prevalence of vascular anomalies in the head and neck region in young patients is almost 60%. Considering their pathophysiology and morphology, vascular lesions are subdivided into vascular tumours and vascular malformations [12].

The first group includes congenital (CHs—congenital haemangiomas) and infantile haemangiomas (His—infantile haemangiomas), which are caused by excessive angiogenesis due to endothelial cell proliferation [10,11,12,13]. They are most common in the head and neck region (65%), followed by the chest area (25%) and the upper or lower limbs (10%) [13]. Infantile haemangiomas manifest weeks to months after birth and are the most prevalent benign tumours in infancy. The spontaneous regression is observed in over 80% of cases and often results in a favourable response to therapy. By contrast, congenital haemangiomas attain their full size at birth and might demonstrate rapid or no regression. The immunohistochemical marker expressed in haemangiomas is Glut-1. The treatment is required in cases where a lesion is adjacent to organs, causing their physiological impairment. The most common treatment for IHs is propranolol, resulting in regression and reduction of residual lesions. Considering that CHs do not respond to pharmacological therapies, the treatment of choice is resectioning [13,14,15].

Vascular malformations are characterized by the impaired maturation of vessels differing in the degree of mesenchymal tissue proliferation. Haemangiomas grow by hyperplasia, whereas vascular malformations grow by hypertrophy. Unlike HMs, vascular malformations are present at birth and do not regress but increase in size with a patient’s age. According to the type of the vessels involved and flow features, there are specified venous (VM 70%), lymphatic (LM 15%), arterio-venous (AVM 6%), and capillary (CM 9%) malformations with slow-flow (VM, LM, CM) or fast- flow properties (AVM) [14,15,16,17]. VMs of the head and neck present diversified clinical features, depending on the type of vascular lesion. Venous malformations (VMs) present as a soft, non-pulsating, compressible lesion. Depending on the depth of the VM, the skin or mucosa might be from a deep purple to bluish or regular colour. These lesions occur in muscles but may also involve skin and mucosa. Areas commonly involved in the head and neck are the masseter, temporalis, tongue muscles, and oral and respiratory mucosa. Soft tissue lesions most commonly occur in the buccal region, followed by the mandibular, sublingual, lingual, and orbital spaces. Also, VMs might be intrabony, with predominance in the mandible [17,18,19,20]. In the presented case, the patient showed facial asymmetry due to the presence of VMs. The neck was enlarged with a dark bluish lesion, which was soft and non-pulsating in palpation. Also, scattered, hard nodules of a dark purple colour were present. The VMs were visible in the lower lip, buccal, and vestibule mucosa. The lesions were not movable in relation to the adjacent tissues. The hypertrophy of sublingual folds was visible. The tongue showed significantly limited mobility. Vascular lesions were located in close proximity to the dental arches of the mandible and maxilla, impeding the regular dental procedures, especially tooth extraction. The slow flow of blood or its stagnation in big venous compartments may result in formation of thrombi, calcifying into the classic “phleboliths”. Phleboliths are intralesional calcifications formed due to venous stasis and inflammation, which are pathognomonic feature of VMs. They are visualized in radiological imaging, especially with compound tomography scans [20,21,22,23,24]. Contrast-enhanced MRI (MRI—Magnetic Resonance Imaging) is considered the most effective imaging modality for establishing or confirming a diagnosis and delineating the extent of and planning therapy for VMs. While computed tomography scanning has a role in determining bony involvement, ultrasonography provides complementary information and may be useful for establishing the difference between a low-flow lesion and a high-flow lesion [22,25,26,27]. In the described case, CT scans revealed areas of densification with calcifications—phleboliths were visible in the skin and subcutaneous tissue of the neck, chin, and cheeks. Among these, dilated vessels were present, suggesting a large vascular malformation.

The treatment of VMs is generally considered to be ineffective, since successful surgery would result in adverse functional and cosmetic outcomes. Thus, the therapy includes regulation of growth, preserving the aesthetic appearance, and decreasing symptoms. Decisions on the treatment methods vary, considering the wide variety of management options and patient presentations. It is recommended that patients are treated in a multidisciplinary centre. Therapy may consist of sclerotherapy, surgical resectioning, laser therapy for superficial VMs—Nd:YAG laser (Nd:YAG—Neodymium-doped Yttrium Aluminum Garnet)—or a combination of these modalities. Small VMs can be excised if well defined, with or without prior sclerotherapy, which helps to delineate the lesion and reduce intraoperative blood loss. Surgical excision remains a viable option for smaller, well-defined lesions, although it can be challenging for larger, less defined venous malformations. In these cases, significant intraoperative bleeding can complicate the identification and preservation of important structures. In general, venous malformations, although benign lesions, can be challenging to diagnose and manage. A multidisciplinary approach is essential in managing these lesions and is suggested as there is no single superior therapy [20,22,27,28,29,30]. A variety of factors, including the size and location of the malformation, may require input from specialists in surgery, radiology, and other fields to determine the optimal treatment.

According to the medical record, the patient was born with venous malformation (“cavernous haemangioma”). During the patient’s childhood, the treatment methods of vascular lesions included X-ray diathermy, implantation of radioactive gold grains, short-term alcohol injections into the tongue (sclerotherapy), and attempts of isolated coagulation. However, regression of the vascular lesions was not observed following any of these therapies. A surgical procedure was performed to remove hypertrophied tongue tissue with the employment of surgical pins to position the tongue.

Patients affected by vascular anomalies in the head and neck area are predisposed to encounter a number of oral health complications, including facial deformity, occlusal dysfunctions, mucosal bleeding, and dental caries. Although haemangiomas are usually diagnosed correctly, other tumours and vascular malformations are likely to be misdiagnosed. Most patients need a multidisciplinary approach including specialists such as an otolaryngologist, oral and maxillofacial surgeon (OMFS—oral and maxillofacial surgeon), and plastic surgeon [29,30,31,32]. Dental treatment of patients with vascular anomalies, especially venous malformations, might be challenging due to the risk of severe bleeding. For these reasons, it is crucial for dentists to address the oral health needs of the patients.

In the presented case, the patient was referred by a general dentist to remove tooth 31, which was adjacent to the VM located in the mucosa of the oral vestibule. Radiological examination revealed the irregular structure of the mandible, which could indicate intraosseous vascular malformation. After clinical and radiological examinations, the decision was made to perform the extraction in a hospital setting due to possible complications. Under local anaesthesia, tooth 31 was removed, and the periapical lesion was sent for immunohistopathological examination. It confirmed purulent granulomatous tissue of an inflammatory aetiology. Although no complications occurred during the surgery, preventively there was a prepared coagulation device and blood units. Healing of the post-extraction socket was uneventful, and the patient did not report severe bleeding. The patient will undergo further conservative treatment. In patients with VMs, it is important to closely monitor oral health to avoid invasive treatment such as tooth extractions, which may result in life-threatening complications. In case of a massive haemorrhage, which might lead to a hypovolemic condition, it is required to administer an immediate intravenous transfusion of red blood cells. A multidisciplinary approach, involving super-selective embolization and surgery after two days, should be considered the treatment of choice. These interventions require a multidisciplinary approach, with hospitalization to ensure adequate management of complications. Patients should be postoperatively controlled to avoid bleeding, bacterial infections, and osteonecrosis [29,30,31,32].

## 4. Conclusions

The presence of extensive vascular malformation in the head and neck area causes difficulties of dental treatment. Due to the risk of possible bleeding, it is necessary to carefully examine and investigate the condition in order to determine the extent of the vascular lesion and to establish an effective treatment plan. In the presented case, the proximity of venous malformation to teeth requiring treatment or extraction was an indication for surgery in a hospital setting. The patient underwent clinical and radiological examinations. The use of CT with intravenous contrast is a valuable method for the assessment of VM extensions that are adjacent to bone structures. The identification of bone erosion on CT scans might indicate bone infiltration by venous malformation, which is important for dental surgical treatment. However, given that CT provides a reduced level of information about blood flow, magnetic resonance imaging is recommended for evaluating VM extensions. In the presented case, an MRI could not be performed due to the metallic surgical pins in the patient’s tongue. Diagnostics include general dentist, oral and maxillofacial surgeon, oncologist, and radiologist cooperation before the final treatment plan. The study’s limitations included the exclusion of other non-invasive methods of radiological imaging, such as Doppler ultrasound or angio-CT, which could have enhanced diagnostic accuracy. Nevertheless, in the presented case, CT scans with contrast allowed for the surgery to be performed without the necessity of further imaging procedures. Despite the absence of observed haemorrhaging, it is important for dental clinics and offices to be equipped with emergency bleeding treatment kits. As the surgical procedure was undertaken in the setting of local anaesthesia, the necessity for postoperative monitoring was negated. Conversely, in surgical interventions requiring general anaesthesia, vital sign monitoring is imperative to ensure patient safety. Although there is no one generally accepted method of treatment, a multidisciplinary approach is necessary to ensure proper dental management of patients with VMs. A comprehensive evaluation of each patient is imperative, including the dimensions, anatomical location, depth, and symptomatology of the VM in relation to the area of dental treatment. This approach is crucial to avoid potential life-threatening complications.

## Data Availability

The data are available upon reasonable request from the corresponding authors.

## Abbreviations

CT—computed tomography, CBCT—cone-beam computed tomography, HM—haemangioma, ECs—Endothelial cells, GLUT-1—Glucose transporter 1, AVM—arterio-venous malformation, VM—vascular malformation, X-ray—X-ray radiation, FDI—Fédération Dentaire Internationale, ISSVA—International Society for the Study of Vascular Anomalies, CHs—congenital haemangiomas, IHs—infantile haemangiomas, LM—lymphatic malformation, CM—capillary malformation, MRI—Magnetic Resonance Imaging, Nd:YAG—Neodymium-doped Yttrium Aluminum Garnet (Laser), OMFS—oral and maxillofacial surgeon.
